# High-sugar diet leads to obesity and metabolic diseases in *ad libitum* -fed rats irrespective of caloric intake

**DOI:** 10.20945/2359-3997000000199

**Published:** 2020-03-04

**Authors:** Daiane Teixeira de Oliveira, Isabela da Costa Fernandes, Graziele Galdino de Sousa, Talita Adriana Pereira dos Santos, Nívia Carolina Nogueira de Paiva, Cláudia Martins Carneiro, Elísio Alberto Evangelista, Natália Rocha Barboza, Renata Guerra-Sá

**Affiliations:** 1 Programa de Pós-graduação em Ciências Farmacêuticas Escola de Farmácia Universidade Federal de Ouro Preto Ouro Preto MG Brasil Programa de Pós-graduação em Ciências Farmacêuticas , Escola de Farmácia , Universidade Federal de Ouro Preto , Ouro Preto , MG , Brasil; 2 Núcleo de Pesquisas em Ciências Biológicas Universidade Federal de Ouro Preto Ouro Preto MG Brasil Núcleo de Pesquisas em Ciências Biológicas , Universidade Federal de Ouro Preto , Ouro Preto , MG , Brasil

**Keywords:** High-sugar diet, energy consumption, Wistar rats, obesity, metabolic diseases

## Abstract

**Objective:**

Provide a comprehensive view of the events surrounding the sugar consumption, under conditions of energy equivalence; through the analysis of behavioral aspects of intake, and of biochemical, metabolic and physiological parameters, as well as the effect of this nutrient on the plasticity of adipose tissue.

**Materials and methods:**

Newly weaned male Wistar rats were classified in two groups and subjected to the following normocaloric diets: standard chow diet or to high-sugar diet (HSD) *ad libitum* for 18 weeks.

**Results:**

The animals submitted to the HSD were associated with a lower caloric intake during the 18 weeks of experimentation. However, the HSD induced a significant increase in body weight, white adipose tissue weight, adiposity index, Lee index, and the levels of triglycerides and very low-density lipoprotein in the serum. In addition, it induced glucose intolerance, insulin resistance and compensatory increase of insulin secretion by pancreatic β-cells. Also increased heart rate and induced hyperplasia, and hypertrophy of retroperitoneal visceral adipose tissue. In the liver, the HSD was associated with increased hepatic lipid content (i.e., triglycerides and cholesterol) and hepatomegaly.

**Conclusion:**

The post-weaning consumption of HSD induces an adaptive response in metabolism; however, such an event is not enough to reverse the homeostatic imbalance triggered by the chronic consumption of this macronutrient, leading to the development of metabolic syndrome, irrespective of caloric intake. These findings corroborate recent evidence indicating that sugar is a direct contributor to metabolic diseases independent of a positive energy balance. Arch Endocrinol Metab. 2020;64(1):71-81

## INTRODUCTION

Compelling evidence supports that the increase in the prevalence of obesity observed during the previous decades is temporally related to the increase in the dietary intake of sugar ( 1 , 2 ). In the previous two centuries, the consumption of sugar increased by approximately 25-fold ( 3 ). Although studies have reported metabolic alterations induced by the consumption of sugar ( 4 - 6 ), the role of this nutrient in the development of obesity and associated diseases is extremely controversial ( 7 , 8 ). The currently available scientific evidence – as observed in review studies and meta-analyses – remains inconclusive. A number of studies have concluded that consumption of sugar is positively associated with components of the metabolic syndrome ( 9 , 10 ). In contrast, other studies have concluded that there is no sufficient evidence to indicate that dietary sugar is harmful to health versus any other source of calories ( 7 , 11 , 12 ).

Previous data from our research group have demonstrated that the intake (4,8 and 12-week feeding period) of a HSD, with adequate energy content, by post-weaning rats induced discrete biochemical changes without necessarily leading to gain of body mass. However, these results were associated with increased adipocyte number and with up-regulation of expression of genes of the pro-adipogenic pathways (Pparg) and down-regulation of anti-adipogenic signals (Wnt signaling components) in retroperitoneal adipose tissue ( 4 ). These findings motivated us to investigate whether this adaptive metabolic reprogramming observed in adipose tissue, during the critical development windows, would be able to preserve metabolic homeostasis (attenuate/inhibit the development of a more pathological phenotype) even during a long period of consumption of HSD. Thus, in the present study, we analyzed the impact of early and sustained exposure to a normocaloric diet with high sugar levels on health. More specifically, we seek to present a broader view of the pathophysiology of metabolic diseases induced by sugar intake as well as elucidate the impact of chronic consumption of this macronutrient on an adaptive response in metabolism.

## MATERIALS AND METHODS

### Animals

Twenty newly weaned (within 21 days) male Wistar rats (weight: 45 ± 5 g) were obtained from the Center for Animal Science of the Federal University of Ouro Preto (UFOP). The animals were housed in cages and maintained under controlled light-dark cycles (12:12 h) and temperature (24±2°C) conditions, with *ad libitum* access to water and food. All the experimental procedures presented in this study were conducted in accordance with the Brazilian guidelines on animal experimentation of the National Council for the Control of Animal Experimentation (CONCEA) and approved by the Committee on Ethics in the Use of Animals of UFOP (protocol number 2014/45).

### Experimental design and composition of diets

The animals were randomly classified into the following two groups: control diet group, fed with standard rat chow (Nuvilab CR1 ^®^ , Colombo, Brazil) (STD, n = 8) which contained 57.16% total carbohydrate (being 0% added sugar), 14.75% fat, 28.09% protein, totaling 308.51 kcal/100 g; and experimental diet group fed with HSD (n = 12) which contained 66.86% total carbohydrate (being 36.32% added sugar), 15.26% fat, 17.89% protein, totaling 286.36 kcal/100 g. The complete composition of the two diets is shown in Table 1 . HSD was composed of 40.45% standard rat chow compacted to powder, 40.45% Moça ^®^ sweetened condensed milk (Nestle, Montes Claros, MG, Brazil), 8.58% crystal sugar, and 10.52% water. Feeding with the respective diets was initiated after weaning and lasted 18 weeks. After the 18 weeks of experimentation, the animals were submitted to fasting for 12:00 hours and euthanized through inhalation of carbon dioxide (exposure to 100% of carbon dioxide gas at a gradual-fill rate of 20-30% of the chamber volume per minute) followed by laparotomy. Blood samples were collected, the serum was separated through centrifugation, and maintained at −80°C until the biochemical analysis was performed. The adipose tissue deposits (i.e., inguinal, retroperitoneal, epididymal, and brown), liver, heart, kidneys, lungs, and muscles (i.e., gastrocnemius and soleus) were dissected and weighed for morphometric assessment. Retroperitoneal adipose tissue fragments were collected in histological cassettes for further processing and preparation of histological slides.

**Table 1:** Composition of experimental diets

Nutrient	Diet g/kg	
	Standard chow	High-sugar
Total Carbohydrate ^1^	440.83	478.63
Added Sugars	0	260.01
Ashes ^1^	68.73	36.27
Crude fiber ^1^	114.27	50.77
Fat ^1^	50.57	48.54
Protein ^1^	216.67	128.07
Moisture ^1^	108.93	257.73
Vitamin A	0.0039	0.0019
Vitamin D _3_	5×10 ^-5^	2×10 ^-5^
Vitamin E	0.051	0.0213
Vitamin K _3_	0.003	0.0012
Vitamin B _1_	0.005	0.0024
Vitamin B _2_	0.006	0.0041
Vitamin B _6_	0.007	0.0030
Vitamin B _12_	2,2×10 ^-5^	1×10 ^-5^
Niacin	0.06	0.0251
Calcium pantothenate	0.02	0.0081
Phosphorus	8.00	4.25
Calcium	10.00	5.22
Folic acid	0.001	0.0004
Biotin	5×10 ^-5^	2×10 ^-5^
Choline	1.9	0.7686
Sodium	2.70	1.46
Iron	0.05	0.0210
Manganês	0.06	0.0243
Zinc	0.06	0.0281
Copper	0.01	0.0041
Iodine	0.002	0.0008
Selenium	5×10 ^-5^	2×10 ^-5^
Cobalt	0.0015	0.0006
Fluorine	0.080	0.0324
Lysine	12.00	4.85
Methionine	4.00	1.62
Total energy (kcal/100 g) ^1^	308.51	286.36
Carbohydrate (% energy) ^1^	57.16	66.86
Fat (% energy) ^1^	14.75	15.26
Protein (% energy) ^1^	28.09	17.89

^1^ The nutritional calculations were performed through bromatological analysis. The other nutrients were calculated based on the manufacturer’s information. “Added sugars” refers to the sum of all monosaccharides and disaccharides (simple carbohydrates) added during the production of the diet, that are not naturally found in the foods.

### Dietary intake and body mass

During the period of dietary intervention, the animals were weighed on a weekly basis to measure the body weight gain. Dietary intake was also determined weekly and the calculations of caloric intake were performed based on the number of kilocalories provided by each diet ( Table 1 ).

### Assessment of blood pressure and heart rate

The blood pressure (i.e., systolic, diastolic, and mean) and heart rate were assessed by the indirect noninvasive method of tail plethysmography (plethysmograph, LE5001, Panlab, Barcelona, Spain). The pressure was determined in millimeters of mercury (mmHg) and the heart rate in beats per minute (bpm). Measurements were performed in the last week of the experiment (week 18) for 4 consecutive days, with five measurements recorded per day. The individual value of the blood pressure was determined based on the average of the readings obtained each day.

### Oral glucose tolerance test (OGTT)

After 18 weeks, the rats were fasted overnight and subjected to an OGTT. Blood samples were collected from the caudal vein at the following time points: 0, 30, 60, 90, and 120 minutes after gavage administration of glucose solution (1 g glucose/kg rat body weight). Glucose levels were measured using the digital glycemic meter Accu-Chek Active ^®^ (Roche Diagnostics GmbH, Mannheim, Germany). The data are presented as the area under the curve.

### Biochemical analyses

The serum concentrations of glucose, total cholesterol, triglycerides, very low-density lipoprotein (VLDL), low-density lipoprotein (LDL), high-density lipoprotein (HDL), creatinine, urea, alanine aminotransferase (ALT), and aspartate aminotransferase (AST) were measured using commercial kits purchased from the Bioclin/Quiabasa laboratory (Belo Horizonte, MG, Brazil) according to the manufacturer’s protocol. The measurements were performed in triplicates using an automated biochemical analyzer Random Access Clinical Analyzer (Wiener Lab, CM 200, São Paulo, Brazil) at the Pilot Laboratory of Clinical Analyses of the School of Pharmacy of the UFOP. The concentration of serum insulin was determined using a Rat/Mouse Insulin ELISA Kit (Millipore, St. Charles, Missouri, USA; Cat.# EZRMI-13K), as recommended by the manufacturer. Homeostatic model assessment for insulin resistance (HOMA-IR) and β-cell function *(* HOMA-β) were calculated as follows: HOMA-IR *=* (fasting insulin mU/L × fasting glucose mmol/L)/22.5 and HOMA-β *=* (20 × fasting insulin mU/L)/(fasting glucose mmol/L − 3.5) ( 13 ).

### Assessment of adiposity

In week 18, the animals were weighed and submitted to the naso-anal length measurement for the determination of the Lee index: body weight (g) ^1^ /naso anal length (cm) ( 14 ). After euthanasia, the white adipose fat pads (i.e., inguinal, retroperitoneal, epididymal) were dissected and weighed to calculate the adipocity index: (sun of weight white adipose fat pads [g] / body weight [g]) × 100 ( 15 ).

### Extraction and quantification of liver lipids

Hepatic lipids were extracted according to the protocol adapted from Folch and cols. ( 16 ). The liver (100 mg) was homogenized using a chloroform/methanol solution (2:1). After vigorous stirring, 400 μL of methanol were added to each sample and the samples were centrifuged for 10 minutes at 3,000 rpm. The supernatant was transferred and homogenized with 800 μL of chloroform and 640 μL of sodium chloride solution (0.73%), followed by centrifugation. The upper phase was gently discarded and the tubes were washed with 600 μL of Folch’s solution (i.e., 3% chloroform solution, 48% methanol, 47% distilled water, and 2% NaCl [0.29%]). This procedure was repeated thrice. The tubes with the extracted lipids were maintained at 40°C until complete evaporation of the solvent. The tubes containing the completely dried lipids were weighed and the extracted hepatic lipid content (%) was calculated as follows: (final tube weight [g] – initial tube weight [g]) / tissue weight [g] × 100. The lipids present in the tubes were resuspended in 500 μL of isopropanol and the extract was used for the biochemical analyses. The measurements of total cholesterol and triglycerides were performed manually using commercial kits purchased from the Bioclin/Quiabasa laboratory (Belo Horizonte, MG, Brazil) according to the manufacturer’s protocol.

### Measurement of adipocyte size and estimation of cell numbers

Retroperitoneal fat pad sections were fixed in methanol-dimethyl sulfoxide solution (8:2), routinely processed, and embedded in paraffin. Histological sections (thickness: 4 mm) obtained using a microtome were stained with hematoxylin & eosin. Sections were microscopically visualized through 40 × objective and 20 randomly selected fields (area of each field = 75.183,8 µm ^2^ ) per slide were digitized using a Leica DFC340 FX microchamber (Leica Microsystems GmbH, Wetzlar, Germany) connected to a Leica DM5000 B microscope (Leica Microsystems GmbH, Wetzlar, Germany). These procedures were performed in the Multiuser Laboratory of Center for Research in Biological Sciences of UFOP. The area of each adipocyte was measured using the ImageJ software (National Institutes of Health, Bethesda, Maryland, USA). The average adipocyte area for each group was obtained from measurements of 40 adipocytes per animal. The number of adipocytes was estimated from the following ratio: retroperitoneal adipose pad [g]/adipose cell volume [cm ^3^ ] × 0.92 ( 17 ).

### Statistical analysis

Statistical analyses were performed using GraphPad Prism, version 6.01 (GraphPad Software, San Diego, CA, USA). The distribution of the data was verified using the Shapiro-Wilk test. Data with normal distribution were analyzed using Student’s t-test and expressed as the mean ± standard deviation of each group. Non-parametric data were analyzed using the Wilcoxon–Mann–Whitney test and expressed as the median (minimum and maximum values) of each group. Differences with p < 0.05 were considered statistically significant.

## RESULTS

### HSD induced changes in food consumption and body composition

**Figure 1:**
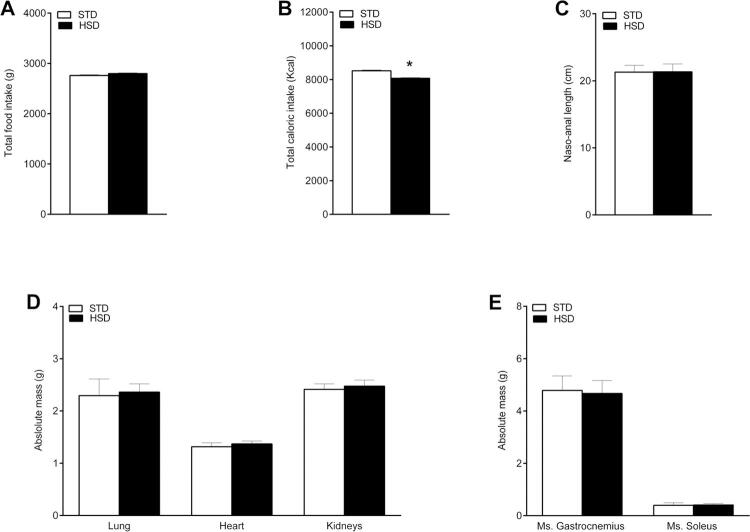
Food consumption and organ mass of rats fed with a standard chow diet (STD) or a high-sugar diet (HSD) for 18 weeks. (A) Total food consumption (g). (B) Total caloric intake (kcal). (C) Naso-anal length (cm). (D) Absolute mass (g) of lung, heart, and kidney. (E) Absolute mass (g) of the gastrocnemius and soleus muscles (Ms.). Data are expressed as the mean ± standard deviation of each group: STD (n = 8) and HSD (n = 12). Data tested using Student’s t-test. * p < 0.05 compared with the STD group. The caloric intake calculations were performed based on the amount of kcal provided by each diet (308.51 kcal/100 g of the STD and 286.36 kcal/100 g of the HSD).

According to our results, the food intake in terms of g ( Figure 1A ) during the 18 weeks of the experiment did not differ between the two groups (p = 0.0785). However, in relation to the total calories ingested ( Figure 1B ), our data reveal a lower caloric intake in the group fed with the HSD (p = 0.0040). Despite the difference in energy intake, the animals exhibited normal development. In addition, the naso-anal length assessment did not demonstrate changes in growth ( Figure 1C ) or the weight of organs ( Figure 1D, 1E ).

**Figure 2:**
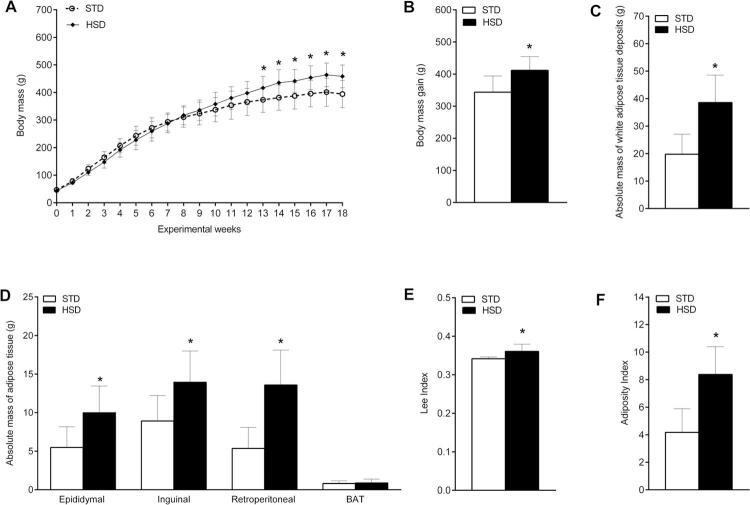
Changes in body composition of rats fed with a high-sugar diet (HSD). ( A ) Weekly body mass gain (g). ( B ) Total body mass gain. ( C ) Absolute total fat mass (g) of white adipose tissue deposits (i.e., the sum of the mass of adipose tissues: epididymal, inguinal, and retroperitoneal). ( D ) Absolute mass (g) of the epididymal, inguinal, retroperitoneal and brown adipose tissue (BAT). ( E ) Lee index. ( F ) Adiposity index. Data are expressed as the mean ± standard deviation of each group: standard chow diet (STD) (n = 8) and HSD (n = 12). Data tested using Student’s t-test. * p < 0.05 compared with the STD group.

As shown in Figure 2A , the HSD induced a greater gain in body mass compared with the STD from week 13 (416.5 ± 41.91 g vs. 373.5 ± 45.49 g, respectively, p = 0.0433). The highest body weight was reached at week 18 (455.2 ± 41.66 g vs. 390.5 ± 51.89 g, respectively, p = 0.0064). Moreover, compared with the STD, consumption of the HSD for 18 weeks induced an increase in body mass (p = 0.0045) ( Figure 2B ) and increased the mass of white adipose tissue deposits (p = 0.0004) ( Figure 2C ). Regarding fat mass, the HSD led to an increase in the epididymal (p = 0.0064), inguinal (p = 0.0094), and retroperitoneal (p = 0.0004) adipose tissues ( Figure 2D ). Furthermore, it increased the Lee index (p = 0.0104) ( Figure 2E ) and adiposity index (p = 0.0006) ( Figure 2F ).

### HSD increased hypertrophy and hyperplasia of retroperitoneal adipose tissue

**Figure 3:**
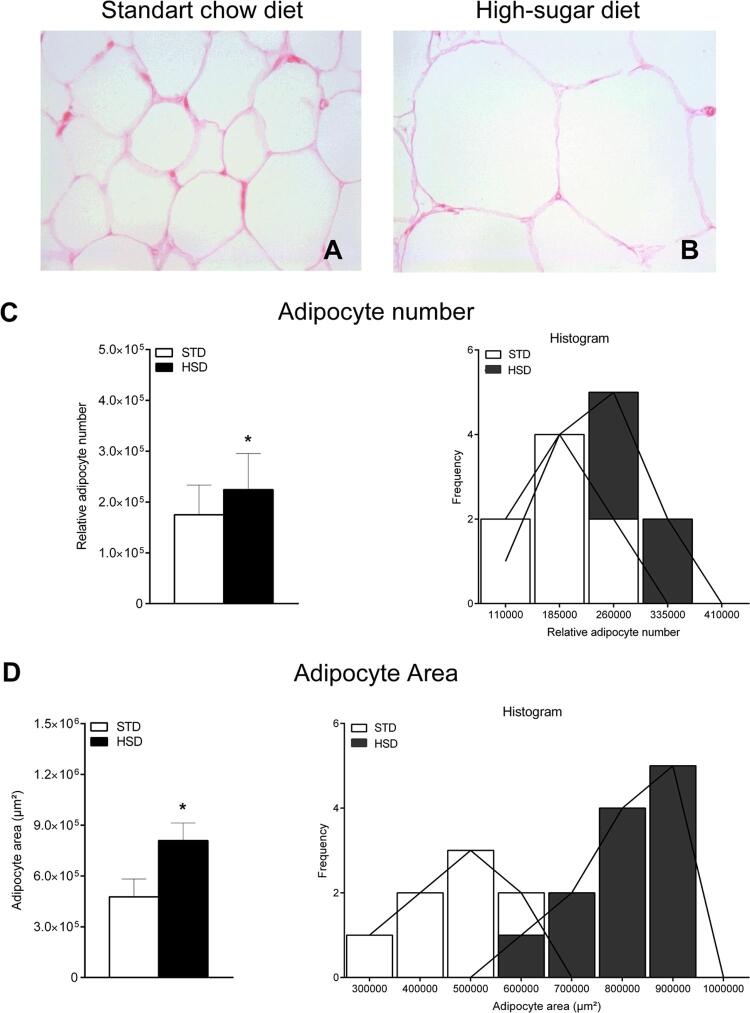
Effect of high-sugar diet (HSD) on the number and area of adipocytes on the retroperitoneal adipose tissue. ( A and B ) Representative photomicrographs of retroperitoneal adipose tissue – histological sections stained with hematoxylin & eosin under × 400 magnification. ( C ) Relative number of adipocytes on retroperitoneal adipose tissue, histogram scale bars: 75000.0. ( D ) Adipocyte area on the retroperitoneal adipose tissue, histogram scale bars: 100000.0. Data are expressed as the mean ± standard deviation of each group: standard chow diet (STD) (n = 8) and HSD (n = 12). Data tested using Student’s t-test. * p < 0.05 compared with the STD group.

Images A and B of Figure 3 represent the histology of retroperitoneal adipose tissue obtained from animals subjected to the STD and HSD, respectively, for 18 weeks. As shown in Figure 3A , the qualitative analyses revealed that the animals subjected to the STD presented normal histology of the retroperitoneal adipose tissue, with the parenchyma predominantly filled by adipose unilocular cells and permeated by the conjunctival stroma. However, consumption of the HSD induced hypertrophy of adipocytes ( Figure 3B ). In addition, the quantitative analyses of photomicrographs of the retroperitoneal adipose tissue revealed that the relative adipocyte number (p = 0.0799) ( Figure 3C ) and adipocyte area (p < 0.0001) ( Figure 3D ) were increased by the HSD. More specifically, the frequency analysis of these data, represented by the histogram, reveals a profile that tends to asymmetry with a skewed left distribution, in comparison to the STD group.

### Metabolic and physiological alterations induced by the HSD

**Figure 4:**
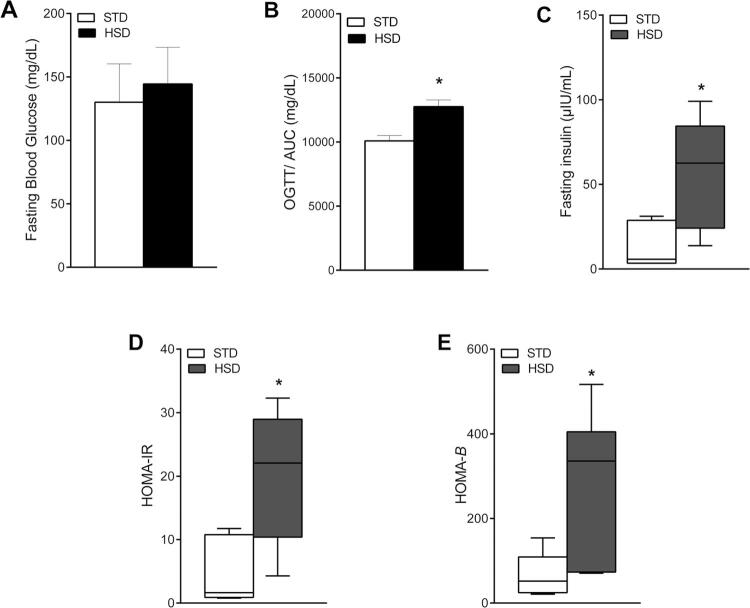
Effects of high-sugar diet (HSD) on insulin sensitivity/resistance. ( A ) Fasting blood glucose (mg/dL). ( B ) Oral glucose tolerance test (OGTT)/area under the curve (AUC) in mg/dL. ( C ) Fasting serum insulin (µIU/mL). ( D ) Homeostatic model assessment for insulin resistance (HOMA-IR). ( E ) Homeostatic model assessment for β-cell function (HOMA-β). Data tested using Student’s t-test or the Wilcoxon-Mann-Whitney test are expressed as the mean ± standard deviation or as the median (minimum and maximum values) of each group: standard chow diet (STD) (n = 8) and HSD (n = 12). * p < 0.05 compared with the STD group.

Data related to the metabolic and vascular profile of the two groups are presented in Table 2 . The results showed that, compared with the STD, the HSD significantly increased the serum levels of triglycerides (p = 0.0007) and VLDL (p = 0.0083). However, it decreased the levels of urea (p = 0.0092). The consumption of the HSD for 18 weeks increased the heart rate compared with the STD. However, the systolic blood pressure, diastolic blood pressure and mean blood pressure were similar in both groups. Furthermore, prolonged intake of the HSD increased the area under the curve in the OGTT (p = 0.0022), serum insulin (p = 0.0077), HOMA-IR |(p = 0.0080), and HOMA-β (p = 0.020) index ( Figure 4 ).

**Table 2:** Metabolic and vascular profile of rats fed with a standard chow diet or a high-sugar diet during the 18-week experiment

Parameters	Standard chow diet (n = 8)	High-sugar diet (n = 12)	p value
	M ± SD / MED	M ± SD / MED	
Cholesterol (mg/dL)	132.62 ± 30.42	136.11 ± 31.21	0.8071
LDL (mg/dL)	67.43 ± 24.31	61.24 ± 30.79	0.6390
VLDL (mg/dL)	16.10 ± 7.81	27.34 ± 8.60*	0.0083
HDL (mg/dL)	47.16 ± 7.35	48.23 ± 5.41	0.7028
Triglycerides (mg/dL)	67.32 ± 18.22	137.55 ± 42.04*	0.0007
AST (U/L)	290.9 (219–336.4)	246.1 (200–630.6)	0.3813
ALT (U/L)	103.23 (77.77–121.1)	105.23 (70.53–525.1)	0.8230
Creatinine (mg/dL)	0.84 ± 0.06	0.89 ± 0.04	0.0557
Urea (mg/dL)	56.96 ± 11.97	44.63 ± 6.19*	0.0092
Heart rate (bpm)	425.80 ± 27.32	384.83 ± 15.96*	0.0013
Systolic pressure (mmHg)	147.78 ± 11.93	140.21 ± 11.20	0.1717
Diastolic pressure (mmHg)	114.87 ± 11. 87	110.14 ± 8.40	0.3440
Mean pressure (mmHg)	126.49 ± 10.14	117.83 ± 8.80	0.0647

Data tested using Student’s t-test or the Wilcoxon–Mann–Whitney test are expressed as the mean ± standard deviation (M ± SD) or median (MED) (minimum and maximum values), respectively. * p < 0.05 compared with the standard chow diet group.

### Effects of the HSD on the accumulation of lipids in the liver

**Figure 5:**
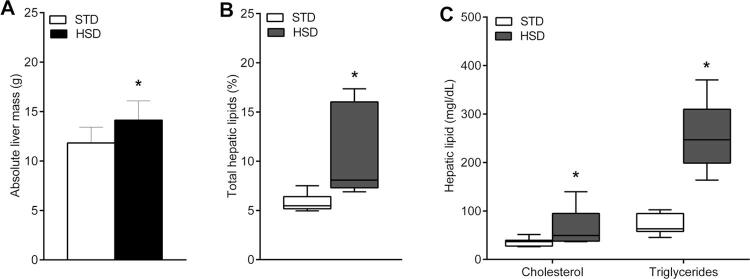
Effects of high-sugar diet (HSD) on the liver. ( A ) Absolute liver mass (g). ( B ) Percentage of hepatic lipid content. ( C ) Biochemical analysis of lipids extracted from the liver (mg/dL). Data tested using Student’s t-test or the Wilcoxon–Mann–Whitney test are expressed as the mean ± standard deviation or as the median (minimum and maximum values) of each group: standard chow diet (STD) (n = 8) and HSD (n = 12). * p < 0.05 compared with the STD group.

As shown in Figures 5A and 5B , the results obtained demonstrate that the prolonged consumption of the HSD led to an increase in the organ mass (p = 0.0123) and lipid content in the liver (p = 0.0002). In relation to lipids extracted from the liver ( Figure 5C ), our results showed increased levels in the livers of rats fed with the HSD versus the STD: triglycerides (258.6 mg/dL vs. 63.02 mg/dL, respectively, p < 0.0001) and cholesterol (49.62 mg/dL vs. 36.75 mg/dL, respectively, p = 0.0298).

## DISCUSSION

The present study provides several levels of evidence that early and prolonged exposure to a HSD leads to obesity and the metabolic syndrome phenotype even in the absence of an increased caloric intake. Firstly, newly weaned rats fed with the HSD exhibited increased body weights and fat deposits of white adipose tissue with hypertrophy and hyperplasia of retroperitoneal visceral fat. Secondly, HSD-fed animals showed increased levels of serum triglyceride and VLDL, glucose intolerance, insulin resistance, increase of the secretory function of the β cells, development of hepatomegaly, and accumulation of hepatic lipids.

Appetite control is not exclusively regulated by the energy balance. Food intake is modulated by several factors, including hormones and metabolites of the biochemical pathways ( 18 , 19 ). Moreover, the composition of the macronutrients included in diets is an important modulator of appetite ( 19 ). According to our data, the animals subjected to the HSD consumed fewer calories compared with those subjected to the STD. This finding corroborates previous data published by our research group showing that the HSD induces an increase in the concentration of leptin – a hormone that acts in the hypothalamus to reduce food intake ( 20 ). In fact, the consumption of sugar modulates the control of appetite through different mechanisms. For example, glucose is the main indicator of the global energy status. Changes in the levels of glucose reflect the energetic state of the cells and modulate key intermediates in the hypothalamic signaling cascade (i.e., AMP-activated protein kinase and malonyl-CoA), which regulate hunger and energy expenditure. In this case, the consumption of a HSD leads to increased levels of glucose and consequently, the production of malonyl-CoA. In turn, hypothalamic malonyl-CoA acts on the signaling of the anorectic-orexigenic neuropeptide system, suppressing the energy intake ( 18 ). Sánchez and cols. also observed that expression of ghrelin (a peptide increasing food intake and body weight) in the stomach was recovered after the administration of a high-fat diet. However, in rats fed with a carbohydrate diet, the levels of ghrelin remained low ( 21 ).

Body weight, fat deposits weight, Lee index, and adiposity index are the biometric parameters used in several studies for the evaluation of obesity in animal models ( 14 , 15 ). Prior to the present research, members of this research group demonstrated that the post-weaning consumption of the HSD for 12 weeks led to increased adiposity without altering body weight and the Lee index ( 4 ). However, in the present study, the HSD induced an increase in body weight (from week 13), white adipose tissue weight, Lee index, and adiposity index. Collectively, these results allow us to infer that time is important in the induction of obesity in the present model of high-sugar dietary manipulation. We emphasize, that the relative total caloric intake and relative mass of adipose tissues maintained the same profile observed in the absolute mass analysis (data not shown). Our results are in agreement with those of other studies showing that the post-weaning exposure of Wistar rats to diets containing excess sugar led to a progressive increase in body weight and/or fat mass, independent of the total energy intake ( 6 , 20 ). As reviewed by Olsen and Heitmann, it appears that sugar intake and the consequent development of obesity is not associated with an increased energy consumption ( 22 ). These findings may be associated with the effects of sugar on insulin signaling and consequent resistance to this hormone and hyperinsulinemia. Since insulin as a lipogenic and anabolic hormone is a direct contributor to increasing body mass ( 23 ).

Increased body fat deposition in obesity may be the result of an increased number of adipocytes (i.e., hyperplasia), increased size of pre-existing adipocytes (i.e., hypertrophy), or both ( 24 ). In childhood obesity, the expansion of white adipose tissue is the result of both hypertrophy and hyperplasia. However, in adults, the number of adipocytes remains fixed and changes in the percentage of fat mass are mainly attributed to the altered adipocyte volume. Thus, the number of adipocytes induced in infancy is a key factor in the accumulation of fat mass in adults ( 25 , 26 ). We previously reported that the use of the HSD and STD induced time-dependent (i.e., at 4, 8, and 12 weeks) hypertrophy in the retroperitoneal adipose tissue. However, in relation to the number of adipocytes, this profile was observed only in animals subjected to the HSD. In addition, these results were associated with the downregulation of adipogenic genes initially observed at the 8-week period in the STD group. However, this observation was not reported in the HSD group, suggesting that this diet modulates at the transcriptional level the anti-adipogenic signals naturally induced by aging ( 4 ). In the present study, we found that post-weaning consumption of an HSD by rats until adulthood also led to hyperplasia and hypertrophy in adipose tissue. Collectively, these results propose that the early and chronic exposure to the HSD is extremely detrimental to health, promoting and sustaining hyperplasia of adipose tissue from the early years of life to adulthood. In addition, it leads to hypertrophy of adipose tissue, irrespective of caloric consumption.

By focusing exclusively on the calories, we may overlook the metabolic effects of each macronutrient. Therefore, it should be noted that the metabolization of macronutrients follows distinct biochemical pathways which may not be equal in energy ( 27 , 28 ). For example, consumption of a high-carbohydrate diet drastically decreases gluconeogenesis (i.e., pathway that consumes four adenosine triphosphates and two guanosine triphosphates). However, it also leads to an increase in glycolysis (i.e., pathway with energy balance of two adenosine triphosphates) ( 29 ). Macronutrients may also produce differences in energy balance through thermogenesis (i.e., thermal effect of feeding). The metabolization of proteins consumes approximately 25%-30% of their energy value, whereas the metabolization of carbohydrates and lipids consumes 6%-8% and 2%-3%, respectively ( 30 ). Considering this, studies have shown that there is a “metabolic advantage” in diets with low carbohydrate content. This type of diet leads to greater weight loss compared with other isocaloric diets of different macronutrient composition ( 28 , 31 ). Consistent with these findings, in the present study, we showed that nutritional quality is more important than the caloric amount and that diet composition exerts an important effect on the development of obesity.

Regarding glycemic control, our results revealed that HSD-fed animals exhibited glucose intolerance, hyperinsulinemia, insulin resistance and increased secretory function of pancreatic β-cells, although fasting glycemia levels were comparable in both groups. Thus, glucose homeostasis observed in this model is marked by the robust plasticity of β-cells (compensatory increase of insulin secretion) in the face of progressive insulin resistance occurring during 18 weeks of HSD consumption. However, this dysfunction is of concern, since the high demand for insulin secretion in response to chronic resistance to this hormone results in the progressive impairment of pancreatic β-cells and consequently the development of type 2 diabetes ( 32 ). Our results indicate that the HSD significantly increases the levels of triglycerides and VLDL in the serum. The relevant literature describes that excess dietary sugar induces the endogenous synthesis of triglycerides through the *de novo* lipogenesis (DNL) pathway, which may lead to increased synthesis and secretion of VLDL lipoprotein into the bloodstream ( 33 ). Relevant for this discussion, insulin resistance is linked with increased secretion of VLDL and increased plasma triglycerides, as well as the development of hepatic steatosis. In states of chronic systemic hyperinsulinemia, the loss of responsiveness to insulin-targeting of apoB for degradation together with increased fatty acid flux to the liver, and increased DNL, results in increased VLDL secretion ( 34 ). In addition, our results showed that the HSD decreased the concentration of urea in the serum compared with the STD. Urea is a metabolite produced by the liver as a result of the metabolization of feed proteins ( 29 ). Therefore, this difference may be attributed to a higher amount of protein present in the STD versus the HSD (28.09% vs. 17.89%, respectively).

As stated by Rippe and Angelopoulos, the effect of sugar consumption on blood pressure remains controversial, with the majority of studies observing this type of interaction in diets with extremely high concentrations of sugars ( 7 ). In our study, there was no statistically significant difference in blood pressure between the groups. However, the HSD induced an increase in heart rate. Lima described that the consumption of a HSD by Wistar rats did not induce changes in blood pressure; however, it induced a reduction in baroreflex sensitivity ( 35 ). In the present study, the HSD led to a 47.61% increase in lipid content in the liver compared with the STD. Ectopic lipid deposition in the liver is strongly characterized as the result of an imbalance between lipid acquisition and elimination of these lipids in the liver. More specifically, such an event can be caused when the uptake of circulating lipids and the de novo synthesis of fatty acids exceed the compensatory capacity of fatty acid oxidation (β-oxidation) and the synthesis and secretion of lipids in VLD in the liver ( 34 ). This type of dysfunction is mainly triggered by insulin resistance ( 34 ).

In conclusion, the present study demonstrated that the early and prolonged consumption of HSD leads to metabolic syndrome phenotype, irrespective of energy intake. This phenotype was triggered even in the face of adaptive responses (hyperplasia and hypertrophy of adipose tissue) to preserve metabolic homeostasis in obesity induced by this diet. These findings contribute to the consolidation of a direct pathway (independent of the positive energy balance) of induction of obesity and metabolic alterations by sugar intake.

## References

[1] Bray GA , Nielsen SJ , Popkin BM . Consumption of high-fructose corn syrup in beverages may play a role in the epidemic of obesity . Am J Clin Nutr . 2004 ; 79 ( 4 ): 537 - 43 . 10.1093/ajcn/79.4.537 15051594

[2] Lustig RH, Schmidt LA, Brindis CD. Public health : The toxic truth about sugar . Nature . 2012 ; 482 ( 7383 ): 27 - 9 . 10.1038/482027a 22297952

[3] Yudkin J. Pure, white and deadly. New York: Penguin Books; 1972 .

[4] de Queiroz KB , Coimbra RS , Ferreira AR , Carneiro CM , Paiva NC , Costa DC , et al . Molecular mechanism driving retroperitoneal adipocyte hypertrophy and hyperplasia in response to a high-sugar diet . Mol Nutr Food Res . 2014 ; 58 ( 12 ): 2331 - 41 . 10.1002/mnfr.201400241 25164976

[5] Malik VS , Hu FB . Sweeteners and Risk of Obesity and Type 2 Diabetes: The Role of Sugar-Sweetened Beverages . Curr Diab Rep . 2012 ; 12 : 195 - 203 . 10.1007/s11892-012-0259-6 22289979

[6] Pinto BAS , Melo TM , Flister KF , França LM , Kajihara D , Tanaka LY , et al . Early and sustained exposure to high-sucrose diet triggers hippocampal ER stress in young rats . Metab Brain Dis . 2016 ; 31 ( 4 ): 917 - 27 . 10.1007/s11011-016-9830-1 27154727

[7] Rippe JM , Angelopoulos TJ . Added sugars and risk factors for obesity, diabetes and heart disease . Int J Obes (Lond). 2016 ; 40 Suppl 1: S22 - 7 . 10.1038/ijo.2016.10 27001643

[8] Stanhope KL . Sugar consumption, metabolic disease and obesity: the state of the controversy . Crit Rev Clin Lab Sci . 2016 ; 53 ( 1 ): 52 - 67 . 10.3109/10408363.2015.1084990 PMC4822166 26376619

[9] Kelishadi R , Mansourian M , Heidari-Beni M . Association of fructose consumption and components of metabolic syndrome in human studies: a systematic review and meta-analysis . Nutrition . 2014 ; 30 ( 5 ): 503 - 10 . 10.1016/j.nut.2013.08.014 24698343

[10] Malik VS , Schulze MB , Hu FB . Intake of sugar-sweetened beverages and weight gain: a systematic review . Am J Clin Nutr . 2006 ; 84 ( 2 ): 274 - 88 . 10.1093/ajcn/84.1.274 PMC3210834 16895873

[11] Kahn R , Sievenpiper JL . Dietary sugar and body weight: have we reached a crisis in the epidemic of obesity and diabetes?: we have, but the pox on sugar is overwrought and overworked . Diabetes Care . 2014 ; 37 ( 4 ): 957 - 62 . 10.2337/dc13-2506 24652726

[12] Kaiser KA , Shikany JM , Keating KD , Allison DB . Will reducing sugar-sweetened beverage consumption reduce obesity? Evidence supporting conjecture is strong, but evidence when testing effect is weak . Obes Rev . 2013 ; 14 ( 8 ): 620 - 33 . 10.1111/obr.12048 PMC3929296 23742715

[13] Matthews DR , Hosker JP , Rudenski AS , Naylor BA , Treacher DF , Turner RC . Homeostasis model assessment: insulin resistance and beta-cell function from fasting plasma glucose and insulin concentrations in man . Diabetologia . 1985 ; 28 ( 7 ): 412 - 9 . 10.1007/BF00280883 3899825

[14] Bernardis LL , Patterson BD . Correlation between ‘Lee index’ and carcass fat content in weanling and adult female rats with hypothalamic lesions . J Endocrinol . 1968 ; 40 ( 4 ): 527 - 8 . 10.1677/joe.0.0400527 4868415

[15] Taylor BA , Phillips SJ . Detection of obesity QTLs on mouse chromosomes 1 and 7 by selective DNA pooling . Genomics . 1996 ; 34 ( 3 ): 389 - 98 . 10.1006/geno.1996.0302 8786140

[16] Folch J , Lees M , Sloane Stanley GH . A simple method for the isolation and purification of total lipides from animal tissues . J Biol Chem . 1957 ; 226 ( 1 ): 497 - 509 . 13428781

[17] Bourgeois F , Alexiu A , Lemonnier D . Dietary-induced obesity: effect of dietary fats on adipose tissue cellularity in mice . Br J Nutr . 1983 ; 49 ( 1 ): 17 - 26 . 10.1079/bjn19830006 6821685

[18] Lane MD , Cha SH . Effect of glucose and fructose on food intake via malonyl-CoA signaling in the brain . Biochem Biophys Res Commun . 2009 ; 382 ( 1 ): 1 - 5 . 10.1016/j.bbrc.2009.02.145 19265677

[19] Carreiro AL , Dhillon J , Gordon S , Higgins KA , Jacobs AG , McArthur BM , et al . The Macronutrients, Appetite, and Energy Intake . Annu Rev Nutr . 2016 ; 36 : 73 - 103 . 10.1146/annurev-nutr-121415-112624 PMC4960974 27431364

[20] de Queiroz KB , Guimarães JB , Coimbra CC , Rodovalho GV , Carneiro CM , Evangelista EA , et al . Endurance training increases leptin expression in the retroperitoneal adipose tissue of rats fed with a high-sugar diet . Lipids . 2014 ; 49 ( 1 ): 85 - 96 . 10.1007/s11745-013-3854-7 PMC3889676 24243000

[21] Sánchez J , Oliver P , Palou A , Picó C . The inhibition of gastric ghrelin production by food intake in rats is dependent on the type of macronutrient . Endocrinology . 2004 ; 145 ( 11 ): 5049 - 55 . 10.1210/en.2004-0493 15284203

[22] Olsen NJ , Heitmann BL . Intake of calorically sweetened beverages and obesity . Obes Rev . 2009 ; 10 ( 1 ): 68 - 75 . 10.1111/j.1467-789X.2008.00523.x 18764885

[23] Laville M, Andreelli F. Mechanisms for weight gain during blood glucose normalization. Diabetes Metab. 2000 ;26 Suppl 3:42-5. 10945152

[24] Choe SS , Huh JY , Hwang IJ , Kim JI , Kim JB . Adipose Tissue Remodeling: Its Role in Energy Metabolism and Metabolic Disorders . Front Endocrinol . 2016 ; 7 :30 10.3389/fendo.2016.00030 PMC4829583 27148161

[25] Christodoulides C , Lagathu C , Sethi JK , Vidal-Puig A . Adipogenesis and WNT signalling . Trends Endocrinol Metab . 2009 ; 20 ( 1 ): 16 - 24 . 10.1016/j.tem.2008.09.002 PMC4304002 19008118

[26] Spalding KL , Arner E , Westermark PO , Bernard S , Buchholz BA , Bergmann O , et al . Dynamics of fat cell turnover in humans . Nature . 2008 ; 453 ( 7196 ): 783 - 7 . 10.1038/nature06902 18454136

[27] Manninen AH . Is a Calorie Really a Calorie? Metabolic Advantage of Low-Carbohydrate Diets . J Int Soc Sports Nutr . 2004 ; 1 ( 2 ):21-6. 10.1186/1550-2783-1-2-21 PMC2129158 18500946

[28] Feinman RD , Fine EJ . Thermodynamics and metabolic advantage of weight loss diets . Metab Syndr Relat Disord . 2003 ; 1 ( 3 ): 209 - 19 . 10.1089/154041903322716688 18370664

[29] Moran LA, Horton HR, Scrimgeour KG, Perry MD. Bioquímica. 5ª ed. São Paulo: Pearson; 2013 .

[30] Jéquier E . Pathways to obesity . Int J Obes Relat Metab Disord J. 2002 ; 26 Suppl 2: S12 - 7 . 10.1038/sj.ijo.0802123 12174324

[31] Bueno NB , de Melo IS , de Oliveira SL , da Rocha Ataide T . Very-low-carbohydrate ketogenic diet v. low-fat diet for long-term weight loss: a meta-analysis of randomised controlled trials . Br J Nutr . 2013 ; 110 ( 7 ): 1178 - 87 . 10.1017/S0007114513000548 23651522

[32] Buchanan TA , Xiang AH , Peters RK , Kjos SL , Marroquin A , Goico J , et al . Preservation of pancreatic beta-cell function and prevention of type 2 diabetes by pharmacological treatment of insulin resistance in high-risk hispanic women . Diabetes . 2002 ; 51 ( 9 ): 2796 - 803 . 10.2337/diabetes.51.9.2796 12196473

[33] Strable MS , Ntambi JM . Genetic control of de novo lipogenesis: role in diet-induced obesity . Crit Rev Biochem Mol Biol . 2010 ; 45 ( 3 ): 199 - 214 . 10.3109/10409231003667500 PMC2874080 20218765

[34] Choi SH , Ginsberg HN . Increased very low density lipoprotein (VLDL) secretion, hepatic steatosis, and insulin resistance . Trends Endocrinol Metab . 2011 ; 22 ( 9 ): 353 - 63 . 10.1016/j.tem.2011.04.007 PMC3163828 21616678

[35] Lima DC. Avaliação do balanço autonômico e da resposta hiperglicêmica à hemorragia em ratos obesos induzidos pela dieta hipercalórica [dissertação]. Belo Horizonte: Universidade Federal de Minas Gerais; 2008 .

